# Barriers to the implementation of a computer-based rehabilitation programme in two public psychiatric settings

**DOI:** 10.4102/sajpsychiatry.v24.i0.1163

**Published:** 2018-06-11

**Authors:** Aline Ferreira-Correia, Tyler Barberis, Lerato Msimanga

**Affiliations:** 1 Department of Psychology, University of the Witwatersrand, South Africa

## Abstract

**Background:**

Working memory (WM) deficits have a negative impact on treatment adherence and quality of life. Efficient and effective interventions are needed in order to improve the cognitive functioning of those affected, especially in low-resource communities. Computer-based rehabilitation programmes (CBRP) are low-cost therapeutic approaches for WM deficits. Perceptions and experiences of target users may influence whether CBRP constitute an effective therapeutic option for adults with cognitive impairment in under-resourced environments.

**Aim:**

The goal of the study was to explore the experiences of a group of volunteers with WM deficits (associated with diagnoses of HIV and schizophrenia), in terms of the perceived barriers they encountered during their participation in a CBRP.

**Methods:**

A qualitative, descriptive research design was implemented. Short interviews and field notes were used in order to investigate the experiences of nine participants in relation to the CBRP. The sample included four participants living with HIV and five with schizophrenia, all with WM deficits.

**Results:**

Using a thematic analysis, eight barriers were identified: unawareness of the cognitive deficit, anticipation of negative results, stigma, difficulties accessing a computer and/or Internet connection, ill health, negative emotional experiences, daily routine challenges and non-conducive or sabotaging environments. A representational model of these barriers is proposed.

**Conclusion:**

The implementation of a cognitive rehabilitation strategy should not only take into consideration issues of access to particular strategies and materials but should also be preceded by an exploration of how individual and contextual barriers are experienced by the potential users, as these contribute to the risk of dropout.

## Introduction

Professionals and researchers in the cognitive rehabilitation arena are dedicated to the creation and advancement of interventions that are efficacious, cost-effective, easily administered and applicable to large numbers of patients across different levels of impairment.^1^ Computer-based rehabilitation programmes (CBRP) are very promising as they offer high levels of customisation, applicability and familiarity.^2^

Many CBRP have been developed under the cognitive retraining paradigm. Although this paradigm has several limitations,^3,4^ its potential has been explored and its efficacy tested in several randomised trials^5^; however, despite the increased attention that these technologies have received, their clinical and financial potential has not yet been clearly established.^6^

The inconsistent findings regarding effectiveness of CBRP have been linked to multiple factors, including characteristics of each diagnostic entity,^7^ the nature of the cognitive function that is targeted by the programme^8^ and practice effects and quantity of the exercise.^9^

The content (in terms of variability and purpose) and design (using the apparatus, adjustment of levels of difficulty and characteristics of the interface) of both software and hardware have been identified as important variables in the implementation and effectiveness of the CBRP, not only in terms of specific technological features but in how this is experienced by users in terms of the complex intersections between the specific types of deficits and the personal characteristics of the users.^10^

Personal variables, including insight into the cognitive deficit,^11^ vulnerability to fatigue,^10^ levels of self- motivation and external support to engage in cognitive rehabilitation^6^ are tied to the degree of success of a particular programme. Lack of engagement is a common challenge in neuropsychological rehabilitation, and promoting a consistent effort is thus of paramount importance, not only for the patients but for all stakeholders around them.^12^

Access (in terms of availability in different contexts, financial costs and familiarity with computers) also plays an important role in the potential efficacy of CBRP.^10^ Having the opportunity to get involved in a rehabilitation programme, being able to afford it and to engage with it at ease are prerequisites for participation in these therapeutic offers.

CogMed is one of the most popular CBRP available, but its potential has, and continues to be, under investigation and debate.^13^ This program claims to provide effective training for working memory (WM), but findings on its effectiveness are inconsistent. These inconsistencies are suggestive of methodological challenges as well as a lack of exploration of the underlying mechanisms that facilitate improvement and transfer effects (or lack thereof) to other WM tasks and other associated cognitive functions.^14^

It is clear that the effectiveness of CBRP, and specifically of CogMed, is currently under scrutiny. However, only two studies of the feasibility of using CBRP in under-resourced environments are available, each one involving paediatric samples in Uganda: one consisting of children with cognitive impairment associated with cerebral malaria^15^ and one involving children with human immunodeficiency virus (HIV).^16^ Both studies found that these types of interventions have significant potential in sub-Saharan Africa, not only because they demonstrated improvements but also because the use of computers and access to Internet in the region is sharply increasing, which facilitates the implementation of these techniques in groups of people with a single facilitator.^15^ Furthermore, despite the challenges posed by difficulties with transport to the clinic and limited prior experience with computers, adherence was good and the treatment was shown to be beneficial.^16^

Overall, CBRP have substantial potential but are associated with a range of barriers^2^ that are perceived and constructed by particular groups in various ways.^17^ Although the literature includes studies testing the rehabilitation potential of these tools, there is a lack of studies of whether these programs are an effective therapeutic option for adults with cognitive impairment in under-resourced environments. Thus, this research aims to examine the participants’ experiences of a CBRP (specifically, CogMed) in terms of the perceived impediments to participation they encountered in the entire process of engagement with the programme. Better understanding of the challenges that are present before and during the implementation of Internet-based cognitive rehabilitation in a low-income context can potentially guide the provision of cognitive therapy in terms of selecting the best match of technology, client and context, as well as informing the support structure required to ensure the best possible outcome for the client.

## Research methods and design

### Study design and data collection

This research employed a qualitative descriptive research design.^18^ This design intends to provide a comprehensive description of participants’ experiences of a phenomenon, without being restricted to a specific interpretative or theoretical framework.^19^

In line with this design, the data collection involved short semi-structured interviews conducted at two points during the research: (1) before commencement of the CBRP and (2) at the end of the rehabilitation, whether this occurred because of completion or attrition.

Two semi-structured interview guides were developed. The pre-intervention guide included questions about the participants’ expectations and concerns with regard to the CBRP and perceptions of potential facilitators and impediments to their engagement with the programme. The post-intervention questionnaire was more comprehensive, including questions eliciting participants’ experiences and perceptions of the content of the program (in terms of the interface, potential cognitive and emotional effects, and levels of difficulty and motivation throughout the process).

Field notes were produced by the researchers during the entire intervention in order to keep track of significant comments made by the participants during the coaching calls, as well as general observations that were deemed potentially significant by the researchers.

### Setting, participants and sampling strategy

Convenience and purposive sampling methods were used. Psychiatrists and nurses at a government mental health hospital and directors of three halfway houses (non-governmental organisations) were approached in order to inform them about the project. These professionals then extended invitations to potential participants and allowed the posting of recruitment ads. Those interested in participating contacted the researchers or requested (via communication with the health professionals) to be contacted telephonically. Meetings were arranged in order to inform them about the research project and to ascertain their suitability for the study. Times and venues for the cognitive assessment and installation of the program were arranged with those who met the inclusion criteria and signed the consent form.

Two diagnostic groups were formed: (1) participants living with HIV and on antiretroviral treatment and (2) participants previously diagnosed with paranoid schizophrenia in full remission. These groups were selected because HIV^19^ and schizophrenia^20^ patients are known to display cognitive deficits associated with the pathology and pharmacological treatment specific to each diagnosis, with WM deficits common to both.^21,22^ Although the neurobiological bases of the WM deficits in HIV and schizophrenia are different,^23,24^ and variances in therapeutic outcomes of CBRP have been linked to the underlying diagnosis,^7^ the implementation of the programme was the same in both groups, thus allowing for the qualitative exploration of the participants’ experiences of the CBRP. Furthermore, participants of both groups were healthcare users of the same government hospital, and therefore it was assumed that the socio-economic status of the participants was relatively homogenous.

A different researcher was assigned responsibility for managing each group in terms of recruiting, interviewing, monitoring and coaching. Those with co-morbid medical and psychiatric diagnoses not related to the main diagnosis were excluded. Only participants with WM impairment (maximum performance in the low average range) and access to a computer and an Internet connection were included. Participants’ demographic characteristics are presented in Table 1.

**TABLE 1:** Sample descriptive characteristics.

Variable	*n*	*M* (s.d.)	Range
Age	9	33.4 (8.41)	26–45
Years of education	9	13.1 (1.16)	12–15
**Gender**			
Females	5	-	55.5%
Males	4	-	44.5%
**Employment status**			
Employed	5	-	55.5%
Unemployed	4	-	44.5%
**Main diagnosis**			
Schizophrenia, paranoid	5	-	55.5%
HIV	4	-	44.5%

s.d., standard deviation.

### Intervention

The CBRP used was the CogMed Working Memory Training Program, a computerised and Internet-based rehabilitation tool. This training consists of 25 daily sessions of approximately 30–40 min each, to be completed in 5 weeks. Each participant carries out the training in their own home. The coach is responsible for providing information about the best training conditions.

The program offers multiple indices that can be used to ascertain the levels of functioning in WM, as well as progress measures including daily total span, daily training and pause times, and performance rate. The interface provides access to detailed quantitative information easily available for the researcher to track the performance on a daily basis. During the program, technical support was provided by the researchers when needed. The researchers monitored daily progress scores, which are automatically registered by the rehabilitation programme. The researchers contacted the participants telephonically once a week in order to provide feedback. If particular problems (such as low or absent engagement in activities) were identified by any of the program’s performance indices, the researcher called more often. These calls also aimed to identify and troubleshoot difficulties and to encourage and congratulate the participant for persistence and achievements.

### Data analysis

Thematic analysis^25^ was used to analyse the data. Themes were defined as pieces of information that were relevant in terms of the research question. Therefore, only references made by the participants (or present in the field notes) in relation to the barriers or obstacles that hindered their participation in the CBRP were extracted. These themes were captured and coded by using an inductive and semantic approach.^26^ This approach is data driven and focuses on meanings explicitly available in the data. Thematic categories were identified based on all the available data, which was produced without establishing an *a priori* principle of saturation. All themes that emerged within the topic were noted and grouped according to their explicit similarities. Subsequently, descriptive labels were developed and then integrated into a flowchart to reflect the interactions between the themes and the period within which they were relevant.

## Ethical consideration

Ethical clearance was obtained from the university. Formal permission was obtained from an HIV outpatient clinic located at a mental health hospital and the halfway houses where the recruitment process took place. Participant information sheets were provided, and consent forms were signed by all participants. Permission from CogMed (http://www.cogmed.com/) to use the computer- and Internet-based rehabilitation programme for working memory for research purposes was requested and obtained. Free licences were granted to all participants.

## Results

The eight barriers described in the following sections were identified as influencing the uptake and adherence to the CBRP.

### Barrier 1: Unawareness of the cognitive deficit

The salience of the cognitive deficit and the awareness of cognitive loss were pivotal factors in participants’ engagement with the programme. Participants who were able to clearly identify cognitive symptoms and take ‘ownership’ of a complaint were more amenable to the suggestion of treatment and perceived the programme as an opportunity for improvement. In contrast, participants who declined the invitation or dropped out of the programme in the first week were typically referred by a healthcare worker who reported cognitive issues in the referral, but the participants themselves did not spontaneously indicate cognitive complaints (despite having shown deficits in previous cognitive assessments). These cases were typically coupled with reports of a lack of understanding about cognition in general and what rehabilitation is (e.g. P1: ‘To tell you honestly, I don’t even know what cognitive training is about!’).

For some participants, rehabilitation was for those with high levels of impairment. This suggests that patients may not have been seeking help with cognitive deficits because they did not see the cognitive deficit as a symptom that could potentially be treated unless it was incapacitating (e.g. P1: ‘I know that my friend went for cognitive training, but she doesn’t really have a memory’).

### Barrier 2: Anticipation of negative results

Expectations such as worsening of anxiety levels because of increased awareness of cognitive symptomatology, or the development of anxiety symptomatology because of exposure to the exercises, were recurrently reported by participants who chose not to participate or who dropped out early. Specifically, participants from the schizophrenia group appeared to take into consideration their abilities to cope with challenges and stress when deciding to do the rehabilitation process, while participants from the HIV group did not seem to have this concern, for example:

‘I’m a bit apprehensive; worried about it being a bit too stressful and a bit … impacting on me negatively and that sort of thing … Like putting too much stress on me, making me feel bad about myself for not being clever enough to figure out the answers.’ (P2)

Two elements of this theme are thus apparent: (1) it is important for a person living with chronic illness to be able to identify emotional triggers in order to avoid relapses; (2) the programme is seen as a potential threat to self-esteem and hence a potential precipitating factor of emotional stress.

### Barrier 3: Stigma

Offering rehabilitation programmes in hospital contexts is often associated with particular services. Attendance at specific clinics may thus force diagnosis disclosure by some healthcare users. This raises important ethical concerns that can act as a barrier to treatment, as a face-to-face invitation compromises the person’s rights to anonymity and privacy. Two of the healthcare workers indicated that their difficulty in attracting participants may have been partly a result of issues of confidentiality in relation to HIV. Additionally, participants who enrolled in the programme suggested that it was difficult to find interested potential users because ‘other people are not as open about disclosing their HIV status’ (Field Notes 1). This was thus an apparent obstacle in the HIV clinic but not for the schizophrenia group.

### Barrier 4: Ill health

Because of the immune suppression that is commonly associated with HIV, even for those patients on antiretrovirals (ARVs), the presence of frequent illnesses can severely interfere with the ability to commit to a time-intensive training programme. Some of the participants were thus unable to enrol for the study because of ill health during the recruitment and installation stage (Field Notes).

### Barrier 5: Difficulties accessing a computer and/or Internet connection

Although one of the inclusion criteria for this research was having access to a computer and Internet connection, in practice, access was problematic. Specifically, the poor quality and high cost of Internet connections interfered negatively with some participants’ motivation to engage in the sessions (e.g. P4: ‘Some days I struggled to connect to the Internet and it made me feel so, so demotivated’). Furthermore, some participants were not aware of the quantity of data that was involved in the use of the program and hence did not plan for the costs involved (e.g. P6: ‘My mom pays for the Internet. I just thought you were going to pay for it’).

The implications of having limited access are threefold. Firstly, the quality of the Internet connection may interfere with the exercises, feedback and monitoring of the program and consequently increase the frustration of participants. Secondly, the amount of data required by the program could become problematic after the participants were involved with the training, which could threaten the participation of some participants with initial high motivation. These factors are out of the control of the participant and appear to negatively affect those with high motivation but low tolerance for frustration, which is a common combination in psychiatric populations. Thirdly, limited access must also be considered as a financial issue, which is of particular concern in contexts of high inequality like South Africa.

Use of the materials was also reportedly affected by the relative ease of access, as participants reported that their motivation was negatively impacted by the effort required to set up the laptop (e.g. P1: ‘I could find the time but I was like, just getting my computer out’). Consequently, some participants reported that these types of interventions would be easier to follow or adhere to if they were easier to access (such as through cellular phones and tablets) and available at times that were more convenient (e.g. P3: ‘But if it was on cell phone, like if it was at a place like where I was at the clinic waiting, then I could’ve done it there’).

### Barrier 6: Negative emotional experiences

Participants who displayed low tolerance for frustration had difficulties in maintaining participation because of the increased difficulty of the exercises. For some participants, the increases in difficulty were challenging and motivating. However, participants with high levels of anxiety associated with low performance reported feeling discouraged and overwhelmed when they experienced failure during the tasks, thus concluding that the programme was beyond their personal capabilities and deciding to abandon it.

Because the program adjusts the level of difficulty according to the progress history of the user, what is initially experienced as successful and rewarding may later on be seen as failure (e.g. P6: ‘I started off doing very well, but I just got worse and worse and worse at it’). The anticipation of failure causes disengagement with the activity and a tendency to give up (e.g. P1: ‘The ones that I couldn’t do, especially near the end, I get like demotivated’).

The increasing difficulty of the exercises also reportedly elicited in some participants a fear of failing (e.g. P1: ‘I suppose in a way I was a bit threatened, in a way. Um, like of failing’) and mental exhaustion (e.g. P3: ‘It was quite draining. It was almost like it took a hell of a lot of energy from me … after I’d done it I felt tired; my mind was completely tired’). These were reported as reasons for dropping out during the middle section of the programme.

### Barrier 7: Daily routine challenges

Participants with difficulties incorporating the programme into their daily routine did not continue the training after initial uptake. Common challenges reported were associated with family and work responsibilities (e.g. P4: ‘There was just no time … It’s difficult to find suitable time during the day at the office’).

Unpredictable schedules or the inability to establish a regular daily training time were recurrently reported as reasons for discontinuance. This issue was particularly present in the schizophrenia group (e.g. P5: ‘My schedule is quite erratic generally so I didn’t have a specific schedule that I kept’).

Participants who were aware of their planning and monitoring difficulties expressed the need for an external source to provide support in this regard (e.g. P2: ‘If I had someone giving me a set timetable’; P1: ‘Say to me: P1 you must do it at ten o’clock today, or P1 you must do it at three’).

### Barrier 8: Non-conducive or sabotaging environment

An ideal environment for the intervention is a quiet, well-lit room without interruptions. In clinical settings the environment is controlled in order to reduce interference. However, all participants engaged in the training in their homes, with many citing frequent interruptions (e.g. noise, cell phone, family members) as hindrances to their motivation to engage with the rehabilitation (e.g. P4: ‘I always have to wait until the kids are sleeping; otherwise they interrupt me a lot and sometimes it’s more difficult to concentrate while they are watching TV’).

The challenge with this tendency arose when the training was dependent on the availability of a facilitative space, instead of implementing a regularly scheduled slot without any regard for when the participant had an optimal cognitive disposition. Hence, participants who dropped out of the programme tended to report that the training was controlled or affected by the environment(s) available to them.

In particular, participants from the schizophrenia group reported dissatisfaction with their training environment on the basis of being disturbed by the presence of other people and a house-related routine that they claimed interfered negatively with their motivation to engage with the programme, for example:

‘Usually what happens is that I have the computer out, and then something will call me away. Like, I must go do this, I must go do that and something like that might stop me … If I go outside there’s people so, and if I’m inside, then someone’s always walking inside.’ (P1)

The environment was also understood as an emotional ambiance, as they reported being negatively affected by the lack of a supportive and encouraging atmosphere (e.g. P9: ‘I just felt this place has, like, breaking me down. People aren’t encouraged … it feels like encouragement or more positivity or more group work or more teamwork’).

## Discussion

**FIGURE 1:**
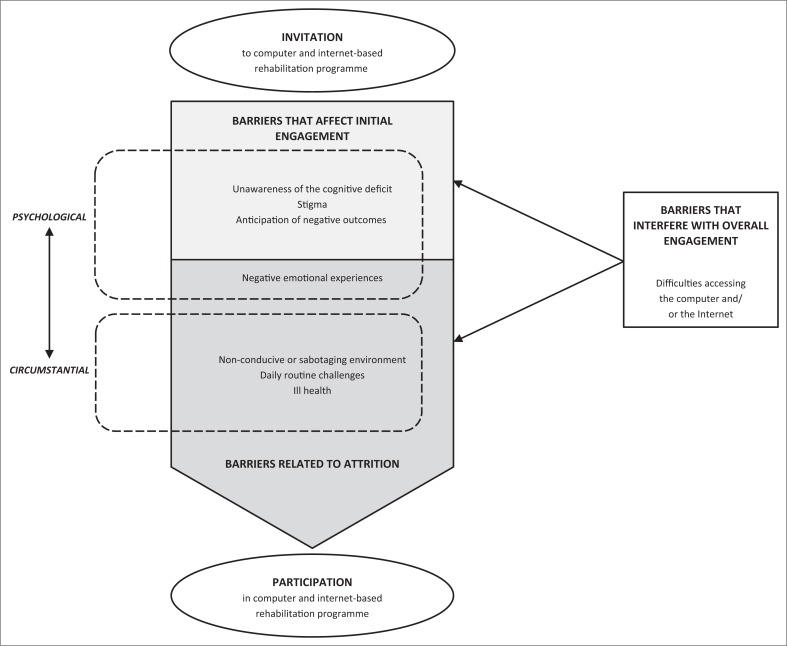
Representational model of the barriers to the implementation of an Internet-based rehabilitation program in a public healthcare setting.

During the implementation of the CBRP, participants experienced several obstacles that were specific to particular moments of the intervention. Figure 1 provides a representation of how participants reportedly experienced impediments from the invitation to take part in the rehabilitation programme to its completion.

Not being aware of the cognitive deficit, experiencing stigma associated with the main clinical diagnosis (HIV or schizophrenia) and anticipating a negative outcome were the three barriers that affected the likelihood of initial participation in the programme. All of them can be understood as psychological variables, as they are cognitive and emotional in nature.

Many psychological variables have been previously identified as obstacles for participation in rehabilitation programmes, with unawareness of the cognitive deficit being prominently linked to lack of participation in cognitive rehabilitation.^26^ In the present study, this issue was twofold. Firstly, not recognising the presence of a cognitive symptom results in no perception of need for treatment, despite the encouragement of a third party (such as the psychiatrist). Therefore, a referral may be futile unless the patient has clearly verbalised a cognitive complaint. Secondly, unawareness of the nature and severity of the cognitive issue and associated therapeutic option was found to reduce participation. Both of these issues are particularly relevant in diagnostic groups where the cognitive impairment is insidious.^27^

The experience of stigma is common in HIV^28^ and schizophrenia.^29^ Although the negative power of stigma could not be fully ascertained in this research, it was observed that there was reduced access to the rehabilitation programme for users of the HIV clinic, which supports the claim that stigma interferes with access to treatment opportunities.^30^

The impact of the anticipation of negative outcomes found in the schizophrenia group was similar to the one previously identified in Alzheimer’s disease, especially in terms of its negative influence on mood and self-efficacy, as well as reluctance to participate in activities that act as triggers for anxiety, depression and low self-esteem.^27^

The theme *negative emotional experiences*, although considered to be also of a psychological nature, had an impact after the initial uptake, which is consistent with previous research reporting that high levels of psychological distress preceding engagement with rehabilitation programmes has been linked to early attrition.^31^ The results suggest that low tolerance to frustration, high anxiety, high vulnerability to mental fatigue and low levels of motivation were associated with attrition. This finding can be interpreted as resulting from cognitive bias, which is common in people living with emotional psychopathology. Cognitive bias makes people more prone to selectively attend to the emotionally negative aspects of an experience.^32,33^

Other barriers that were frequently reported as reasons to give up the CBRP after the set-up session seemed to be circumstantial. These themes were presented as situations that were outside the participants’ control. An example is daily routine challenges, which interfered with the scheduling of the rehabilitation session. This difficulty incorporating the programme into the routine and maintaining a consistent practice is possibly related to deficits in other cognitive domains, such as executive functions. Research has interpreted the failure to incorporate activities into the daily schedule, as well as sourcing a conducive environment, as symptoms of executive dysfunction^34,35^ that are common in people with schizophrenia^36,37^ and HIV.^38,39^

A central issue for the participants was associated with their living conditions, which were reported in some cases to be non-conducive or even sabotaging. The lack of an appropriate space that provided privacy, silence and encouragement, or at least did not actively interfere with the training sessions, was an important reported reason for attrition. Despite participants being informed about the ideal environmental conditions for training (such as good light, comfortable seating and no interruptions), most of them were unable to work under these conditions. This is a strong indicator that take-home rehabilitation programmes may be problematic, thus therapists should weigh the challenges associated with going to the hospital^15^ against the benefits of controlling the rehabilitation environment.

Having ill health can be a circumstantial barrier when mild or acute conditions coincide with the beginning of the programme. Previous research has reported medical conditions as a reason for withdrawing participation.^40^ When users have a chronic health condition that makes them vulnerable to health problems, such as HIV, it is possible that the perception of one’s health can be used as a reason to refrain from participating in long commitments that require daily engagement, such as CBRP.

Limited access to the computer or Internet affected participation at all stages of implementation. This particular theme was associated with psychological and circumstantial factors. In the first instance, this obstacle reportedly triggered feelings of frustration or conflict with important stakeholders, such as family members. Furthermore, personal variables, such as lack of motivation, reduced energy and low tolerance to frustration, were expressed in relation to managing access (e.g. setting up the workstation or laptop was reportedly an extraordinary effort), which connects the theme of limited access with the cognitive bias and dysexecutive syndrome described above.

Difficulties accessing a computer and/or Internet connection appeared to be linked to circumstantial factors, specifically to the financial resources available and program accessibility. User experience in cognitive rehabilitation is an important factor for consideration. Programs that are not error-free and require significant computer skill or maintenance from users are likely to exhibit decreased efficacy.^10^

The quality and costs of the Internet connection were particularly relevant. Better quality connections are usually more expensive, and programs that utilise the Internet also require resources in terms of bandwidth, which results in additional expenses to participants. This factor is significant when considering the socio-economic status (and financial dependence) of participants in any intervention. Interventions that are unable to plan for and address unique environmental or social factors in their implementation may face problems with recruitment or compliance. Financial affordability is a serious concern in neuropsychological rehabilitation in general, and more so in low-income contexts.

## Limitations

Several limitations associated with the methodology implemented should be acknowledged. First, the qualitative design only provides a descriptive level of analysis, with low potential for generalisability to other contexts and other diagnoses.^41^ Although purposive, non-probabilistic sampling is typically used in qualitative research,^42^ it is possible that the type of intervention (computer-based) as well as the method of recruiting could have introduced a selection bias, as many of the participants attending the health centres did not have a computer or did not attend consultation with their healthcare practitioner during the recruitment phase. Efforts to minimise researcher bias and improve the validity of the analysis were taken, including additional reviewers during the data analysis process; however, the use of field notes may have resulted in a bias in the presentation of results relating to the researchers’ identification of what was important enough to include in the notes.

## Conclusion

Our findings suggest that participants faced several obstacles that hindered their participation in the CBRP. The experience of stigma, a lack of awareness of the cognitive deficit and the anticipation of negative outcome were barriers that apparently interfered with the initial engagement with the treatment. Having negative emotional experiences associated with the programme, experiencing the environment as non-conducive or sabotaging of the rehabilitation, facing daily challenges and ill health seemed to be linked to an increased risk of attrition after the CBRP had begun. Limited access to a computer and the Internet reportedly reduced the chances of participation at all stages of the CBRP.

These findings suggest that, prior to the selection of a cognitive rehabilitation option and recommendation to specific candidates, the healthcare professional should take into consideration not only issues of access to particular strategies and materials but also individual and contextual factors that may pose a higher risk for treatment dropout, such as the ones highlighted here. Understanding how these barriers to participation in particular therapeutic options are experienced and perceived by users is of paramount importance in order to select better therapeutic strategies as well as to increase the chances of treatment uptake and adherence.
